# Dataset for transcriptional response of barley (*Hordeum vulgare*) exposed to drought and subsequent re-watering

**DOI:** 10.1016/j.dib.2016.05.051

**Published:** 2016-05-30

**Authors:** Filip Kokáš, Petr Vojta, Petr Galuszka

**Affiliations:** Department of Molecular Biology, Centre of the Region Haná for Biotechnological and Agricultural Research, Faculty of Science, Palacký University in Olomouc, Czech Republic

**Keywords:** Barley, Drought stress, Genome annotation, Re-watering, Transcriptomics

## Abstract

Barley (*Hordeum vulgare*) is an economically important species, which can be cultivated in environmentally adverse conditions due to its higher tolerance in contrast to other cereal crops. The draft of *H. vulgare* genome is available already for couple of years; however its functional annotation is still incomplete. All available databases were searched to expand current annotation. The improved annotation was used to describe processes and genes regulated in transgenic lines showing higher tolerance to drought in our associated article, doi:10.1016/j.nbt.2016.01.010 (Vojta et al., 2016) [1]. Here we present whole transcriptome response, using extended annotation, to severe drought stress and subsequent re-watering in wild-type barley plants in stem elongation phase of growth. Up- and down-regulated genes fall into distinct GO categories and these enriched by stress and revitalization are highlighted. Transcriptomic data were evaluated separately for root and aerial tissues.

Specifications Table

**Table:** 

Subject area	Biology
More specific subject area	RNA-seq transcriptome data of barley (*Hordeum vulgare*)
Type of data	Tables and figures
How data was acquired	Sequencing on Illumina HiSeq 2500 Sequencing System
Data format	Processed, analyzed
Experimental factors	Samples were exposed to severe drought stress and subsequently re-watered
Experimental features	RNA was extracted using RNAqueous kit and purified on magnetic beads. Sequencing libraries were prepared using the TruSeq Stranded mRNA kit from Illumina and quantified using the Kapa Library Quantification kit. Libraries were sequenced on HiSeq 2500 Illumina platform.
Data source location	Palacký University, Olomouc, Czech Republic
Data accessibility	Data are within this article

## Value of the data

• Improvement of a functional annotation of *Hordeum vulgare* genome draft.
• This dataset provides the list of all up- and down-regulated genes during one day long desiccation and subsequent re-watering separately in roots and upper part of 4-week-old barley seedlings.
• Enriched gene ontology (GO) term analysis highlights processes targeted by above mentioned conditions.
• The dataset can serve as a source of candidate genes for markers used for drought associated studies.

## 1. Data

**Fig. 1:**
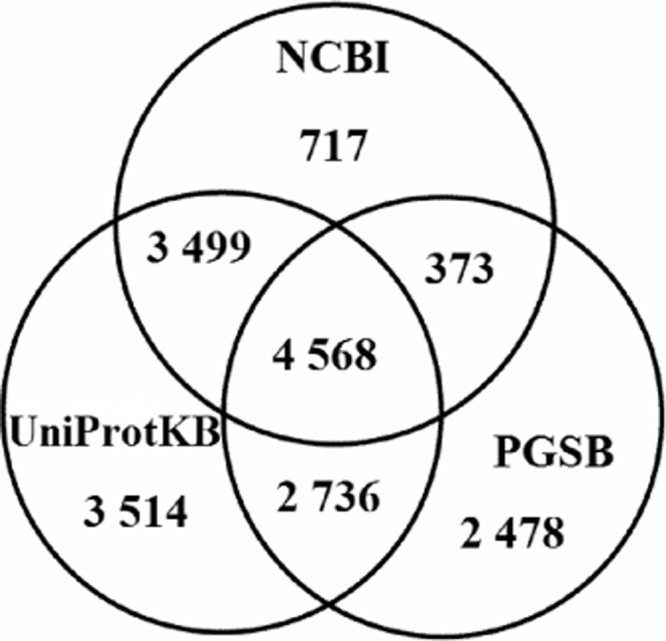
Venn diagram showing numbers of genes due to the source database used for their annotation.

**Fig. 2:**
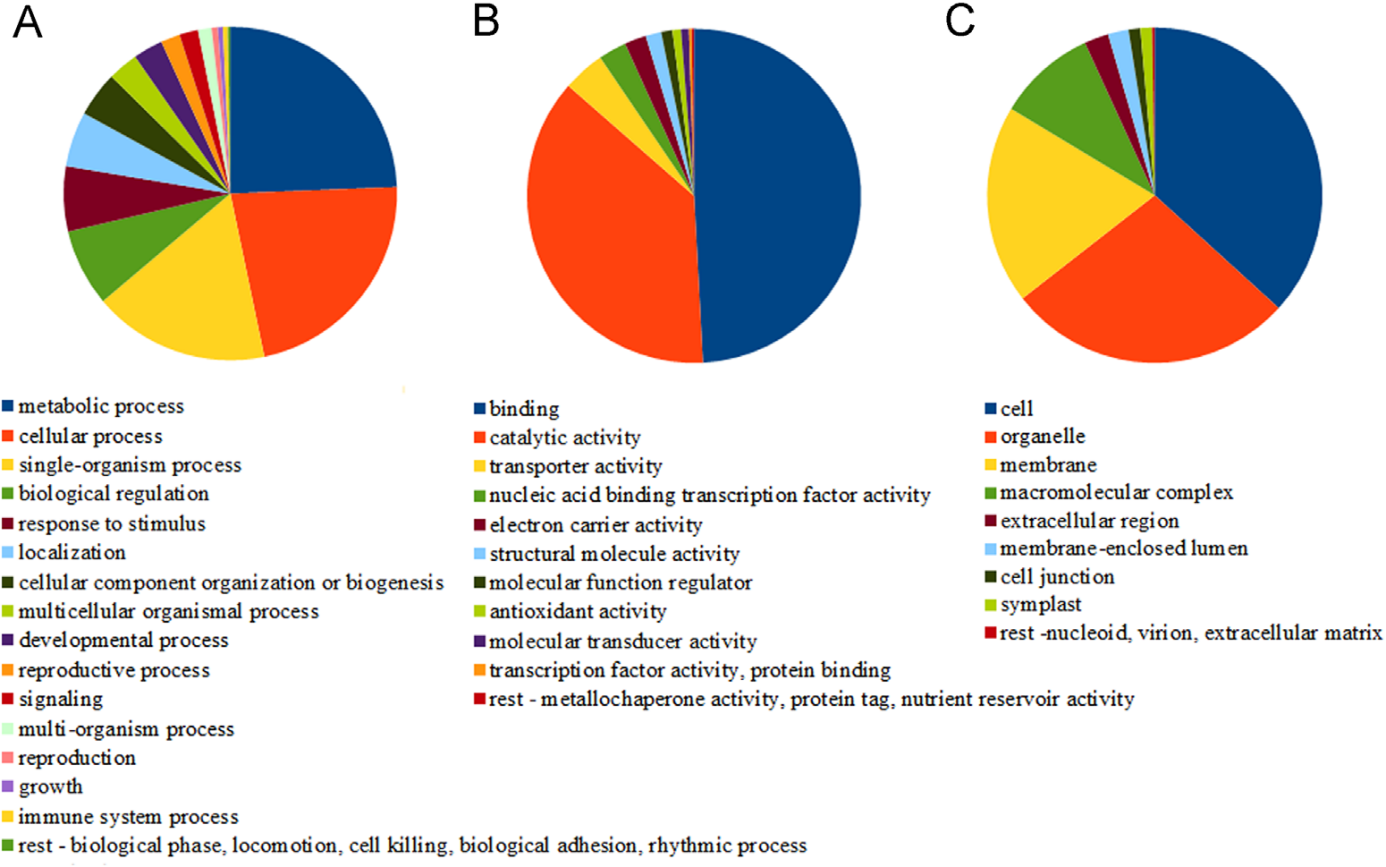
Distribution of GO terms in whole transcriptome on the level 2 for Biological Processes (A), Molecular Function (B) and Cellular Component (C).

**Fig. 3:**
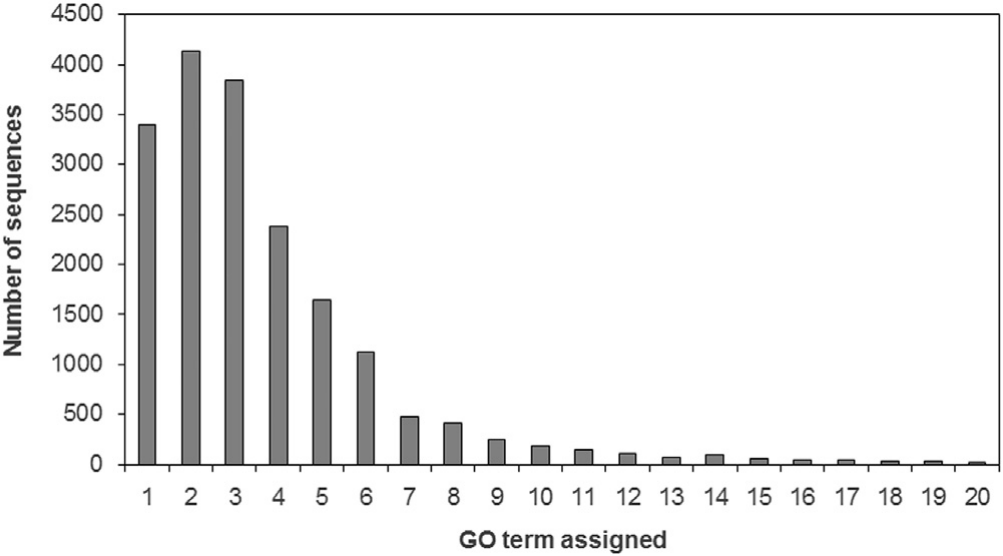
GO terms distribution per sequence annotated in improved *Hordeum vulgare* reference genome.

This data consist of five high-throughput sequenced samples of barley roots (Supplementary Table 1, *n*=2) and upper part (Supplementary Table 2, *n*=3), exposed to optimal or drought conditions and subsequent re-watering, generated from an Illumina HiSeq 2500, together with GO term analysis of the most affected Biological Processes (Table 1, Table 2, Table 3). Predicted genes from the latest genome version (082214v1.25) have been annotated based on three various databases (Fig. 1) and associated to GO term categories (Fig. 2). Several GO terms have been assigned to each predicted sequence (Fig. 3).

## 2 Experimental design, materials and methods

### 2.1 Plant material

Spring barley plants, cultivar Golden Promise, were grown in a phytotron with a photoperiod of 15 °C/16 h light and 12 °C/8 h dark in soil or in hydroponic tanks containing aerated Hoagland nutrient solution. Samples of root tissue 4 weeks after germination were collected from hydroponically grown plants due to the inability to collect root tissues from soil without initiation of mechanical stress. The stress was applied by removing the nutrient solution off the tank. Control samples were collected just before stress induction; stressed root samples were collected 24 h later. Aerial part samples were collected from 4 week old plants cultivated in the shallow soil. Watering on daily basis was interrupted for four days and stressed samples were collected in the end of the drought period. Revitalization samples were collected 12 h after re-watering. Each sequencing library was prepared from pool of 3 individual plants.

### 2.2 Annotation

Additional annotation of predicted genes was mined using Blast2GO version 3.0 program to improve raw reference genome available at Ensembl (http://plants.ensembl.org/index.html, version 082214v1.25). Gene description from the National Center for Biotechnology Information database (NCBI; version b2g_Jan15) were mined using the BLAST module from program Blast2GO with parameters blastn and e-value ≤10^−5^. The other step in annotation process was mapping predicted genes to other databases using Blast2GO with default parameters. Additional annotation of other predicted genes was extracted from The UniProt Knowledgebase database (http://www.uniprot.org/, version 2015_02) and the Plant Genome and Systems Biology database (PGSB; http://pgsb.helmholtz-muenchen.de/plant/, version 2014_07_31) for hits with blastn stringency of e-value ≤10^−5^. Finally, annotation information was obtained for 17,885 genes from a total number of 26,072 predicted genes in *Hordeum vulgare* genome (Fig. 1).

Gene ontology analysis was performed using the Blast2GO v.3.0 [2], firstly for all predicted genes and then specifically for significantly up-regulated and down-regulated genes with adjusted *p*-value (padj) ≤0.05. Total number of 70,719 GO terms was assigned to 20,991 predicted genes. Out of these 40.87%, 42.12% and 17.01% were assigned to Biological Processes, Molecular Function and Cellular Component GO categories, respectively (Fig. 2). Number of GO terms assigned to one predicted sequence was in range from 1 to 35 (Fig. 3). Differentially expressed genes were categorized to Biological Processes (BP), Cellular Components (CC) and Molecular Functions (MF) on the level 6. Number of differentially expressed genes for particular GO terms was compared with total number of genes assigned to the term and enriched GO terms were highlighted (Table 1, Table 2, Table 3). Supplementary Appendix A, Appendix A contains GO terms at the level 6 with associated 10 or more genes in roots and aerial part, respectively. GO terms with associated 9 or less genes were filtered out and are not listed. GO terms are sorted due to increased percentage in category of differentially expressed genes with adjusted *p*-value ≤0.05 from total number of genes with the same assigned GO term. The 30 most affected Biological Processes are shown for stressed root (Table 1), stressed aerial part (Table 2) and the aerial part after re-watering (Table 3).

### 2.3 RNA-extraction and sequencing

Total RNA was extracted and cDNA sequencing library was prepared and sequenced as described elsewhere [1], [3].

### 2.4 RNA-seq analysis

**Fig. 4:**
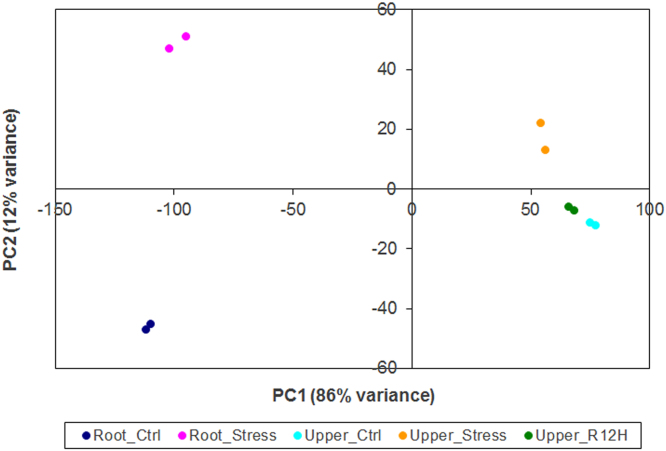
The PCA analysis for replicates from root samples before stress (Root_Ctrl) and during drought stress period (Root_Stress), and from the upper part before stress (Upper_Ctrl), during stress (Upper_Stress) and 12 h after re-watering (Upper_R12H).

Single end reads generated by the sequencing were mapped to the reference genome and quantified the same way as described in Ref. [1]. The comparison for differentially expressed genes among 3 time-points (before stress, during stress, 12 h after re-watering) was conducted using the DESeq2 package [4] implemented in R (R Development Core Team, 2008). Normalized RPKM (reads per kilobase of transcript per million reads mapped) were subjected to principle components analysis (PCA) in order to control quality of replicates. The PCA analysis shows good accordance between replicates, which cluster together (Fig. 4). The log2fold value is calculated for each gene and genes are sorted according to adjusted *p-*value. Positive log2fold values are for up-regulated and negative for down-regulated genes. The base mean value represents mean of normalized RPKM for all comparisons and thus expresses transcript abundance of each gene in particular organ.

## Figures and Tables

**Table 1:** The most affected GO terms from Biological Processes in the stressed roots and percentage of differentially expressed genes (adjusted *p-*value ≤0.05) at the GO level 6.

**GO number**	**GO term**	**Total #**	**% of affected genes**
**DOWN-REGULATED**			
GO:0010089	xylem development	13	69.23%
GO:0071103	DNA conformation change	104	59.62%
GO:0070726	cell wall assembly	11	54.55%
GO:0048544	recognition of pollen	91	50.55%
GO:0006915	apoptotic process	296	50.34%
GO:0051129	negative regulation of cellular component organization	10	50.00%
GO:0001666	response to hypoxia	16	50.00%
GO:0009664	plant-type cell wall organization	76	48.68%
GO:0046271	phenylpropanoid catabolic process	21	47.62%
GO:0007166	cell surface receptor signaling pathway	42	47.62%
GO:0009834	plant-type secondary cell wall biogenesis	19	47.37%
GO:0015851	nucleobase transport	13	46.15%
GO:0006002	fructose 6-phosphate metabolic process	11	45.45%
GO:0042886	amide transport	65	44.62%
GO:0000910	cytokinesis	106	43.40%

**UP-REGULATED**			
GO:0071462	cellular response to water stimulus	11	63.64%
GO:0009407	toxin catabolic process	33	60.61%
GO:0072348	sulfur compound transport	10	60.00%
GO:1902644	tertiary alcohol metabolic process	22	59.09%
GO:0044242	cellular lipid catabolic process	97	57.73%
GO:0033015	tetrapyrrole catabolic process	35	57.14%
GO:0042538	hyperosmotic salinity response	20	55.00%
GO:0010286	heat acclimation	26	53.85%
GO:0046164	alcohol catabolic process	10	50.00%
GO:0046434	organophosphate catabolic process	12	50.00%
GO:0048545	response to steroid hormone	20	50.00%
GO:0050801	ion homeostasis	80	48.75%
GO:0055082	cellular chemical homeostasis	52	48.08%
GO:0042542	response to hydrogen peroxide	62	46.77%
GO:0009699	phenylpropanoid biosynthetic process	28	46.43%

**Table 2:** The most affected GO terms from Biological Processes in the stressed aerial part and percentage of differentially expressed genes (adjusted *p-*value ≤0.05) at the GO level 6.

**GO number**	**GO term**	**Total #**	**% of affected genes**
**DOWN-REGULATED**			
GO:0009765	photosynthesis, light harvesting	32	87.50%
GO:0019750	chloroplast localization	67	71.64%
GO:0051667	establishment of plastid localization	67	71.64%
GO:0009668	plastid membrane organization	123	70.73%
GO:0009658	chloroplast organization	146	67.12%
GO:0016226	iron-sulfur cluster assembly	70	62.86%
GO:0019682	glyceraldehyde-3-phosphate metabolic process	216	62.04%
GO:0051156	glucose 6-phosphate metabolic process	121	61.16%
GO:0033014	tetrapyrrole biosynthetic process	102	59.80%
GO:0042727	flavin-containing compound biosynthetic process	12	58.33%
GO:0010374	stomatal complex development	69	53.62%
GO:0009767	photosynthetic electron transport chain	57	50.88%
GO:0006720	isoprenoid metabolic process	255	49.02%
GO:0006778	porphyrin-containing compound metabolic process	138	47.83%
GO:0016143	S-glycoside metabolic process	60	46.67%

**UP-REGULATED**			
GO:0042538	hyperosmotic salinity response	20	50.00%
GO:0009962	regulation of flavonoid biosynthetic process	11	36.36%
GO:0010647	positive regulation of cell communication	15	33.33%
GO:0006026	aminoglycan catabolic process	18	33.33%
GO:0046348	amino sugar catabolic process	18	33.33%
GO:1901071	glucosamine-containing compound metabolic process	18	33.33%
GO:0060548	negative regulation of cell death	29	31.03%
GO:0010583	response to cyclopentenone	14	28.57%
GO:0046271	phenylpropanoid catabolic process	21	28.57%
GO:0033015	tetrapyrrole catabolic process	35	28.57%
GO:0009414	response to water deprivation	68	27.94%
GO:1902644	tertiary alcohol metabolic process	22	27.27%
GO:0043067	regulation of programmed cell death	63	25.40%
GO:0006662	glycerol ether metabolic process	32	25.00%
GO:0009407	toxin catabolic process	33	24.24%

**Table 3:** The most affected GO terms from Biological Processes in the aerial parts 12 h after re-watering and percentage of differentially expressed genes (adjusted *p-*value ≤0.05) at the GO level 6.

**GO number**	**GO term**	**Total #**	**% of affected genes**
**DOWN-REGULATED**			
GO:0009765	photosynthesis, light harvesting	32	81.25%
GO:0071462	cellular response to water stimulus	11	63.64%
GO:0051156	glucose 6-phosphate metabolic process	121	53.72%
GO:0009767	photosynthetic electron transport chain	57	52.63%
GO:0019750	chloroplast localization	67	50.75%
GO:0051667	establishment of plastid localization	67	50.75%
GO:0072525	pyridine-containing compound biosynthetic process	20	50.00%
GO:0009637	response to blue light	49	48.98%
GO:0019682	glyceraldehyde-3-phosphate metabolic process	216	46.30%
GO:0010109	regulation of photosynthesis	13	46.15%
GO:0043085	positive regulation of catalytic activity	79	45.57%
GO:0016143	S-glycoside metabolic process	60	45.00%
GO:0006778	porphyrin-containing compound metabolic process	138	44.93%
GO:0009668	plastid membrane organization	123	44.72%
GO:0033014	tetrapyrrole biosynthetic process	102	43.14%

**UP-REGULATED**			
GO:0042273	ribosomal large subunit biogenesis	14	71.43%
GO:0000741	karyogamy	25	64.00%
GO:0072528	pyrimidine-containing compound biosynthetic process	97	59.79%
GO:0000085	mitotic G2 phase	23	56.52%
GO:0043572	plastid fission	11	54.55%
GO:0006518	peptide metabolic process	498	49.20%
GO:0043604	amide biosynthetic process	512	48.44%
GO:0007292	female gamete generation	69	46.38%
GO:0007006	mitochondrial membrane organization	11	45.45%
GO:0051604	protein maturation	47	44.68%
GO:0006026	aminoglycan catabolic process	18	44.44%
GO:0046348	amino sugar catabolic process	18	44.44%
GO:1901071	glucosamine-containing compound metabolic process	18	44.44%
GO:0051169	nuclear transport	125	44.00%
GO:0009553	embryo sac development	110	43.64%

## Appendix A Supplementary material

**Table S1.** Comparative transcriptomics of *Hordeum vulgare* roots during the drought stress. Average expression level (baseMean) and change due to optimal conditions (log2FoldChange) with statistical significance (padj) are presented. **Table S2.** Comparative transcriptomics of *Hordeum vulgare* aerial part during the drought and 12 h after re-watering. Average expression level (baseMean) and change due to optimal conditions (log2FoldChange) with statistical significance (padj) are presented. **Table S3.** GO analysis at the level 6 of differentially expressed genes (adjusted *p-*value ≤0.05) in *Hordeum vulgare* roots during the drought stress. Data are categorized according to Biological Processes (BP). Molecular Function (MF) and Cellular Component (CC) and sorted by percentage of affected genes. **Table S4.** GO analysis at the level 6 of differentially expressed genes (adjusted *p-*value ≤0.05) in *Hordeum vulgare* aerial part during the drought and 12 h after re-watering. Data are categorized according to Biological Processes (BP). Molecular Function (MF) and Cellular Component (CC) and sorted by percentage of affected genes.

## Footnotes

Appendix A Supplementary data associated with this article can be found in the online version at 10.1016/j.dib.2016.05.051.
